# Considerations for Menstrual Suppression in Patients With Hematologic Malignancies

**DOI:** 10.1002/phar.70114

**Published:** 2026-02-09

**Authors:** Sahaana Veeravalli, Allison Schepers, Lydia L. Benitez, Martina S. Fraga

**Affiliations:** ^1^ Department of Pharmacy University of Michigan Health Ann Arbor Michigan USA

**Keywords:** hematologic, malignancy, menstrual suppression, menstruation, prophylaxis

## Abstract

Patients who menstruate during treatment for hematologic malignancies have a higher risk of heavy vaginal bleeding due to thrombocytopenia caused by malignancy and myelosuppressive chemotherapy. Heavy menstrual bleeding is associated with significant morbidity in this patient population, and menstrual suppression is a standard of supportive care. Options for prophylactic menstrual suppression include gonadotropin‐releasing hormone agonists, progestin‐only therapy, and combined hormonal contraception. Various factors impact the choice of menstrual suppression agent, including efficacy, presence of emergent acute uterine bleeding, desire for contraception, and presence of thrombotic risk factors. The purpose of this review is to discuss the nuances of available menstrual suppression agents in patients with hematologic malignancies and develop a methodical approach to consider patient‐specific factors that impact agent selection.

## 1 Introduction

Menstrual suppression is an increasingly recognized component of care for patients who are diagnosed with hematologic malignancies. Patients who menstruate largely fall into the adolescent and young adult (AYA) population which includes patients aged 15–39 years old [1]. Acute leukemias and lymphomas are the most frequent hematologic malignancies to occur in this age group. Hodgkin and non‐Hodgkin lymphoma have a 5‐year incidence of 3.4 per 100,000 persons and acute leukemias have a 5‐year incidence of 3.1 per 100,000 persons between 2018 and 2022 in this patient population [2]. Treatment for these cancers typically consists of intensive chemotherapy courses which result in periods of thrombocytopenia and increased bleeding [3, 4]. Heavy vaginal bleeding in this population causes rapid platelet consumption, potentially necessitating increased platelet and red blood cell (RBC) transfusions along with the associated risks of alloimmunization and infection [5]. This highlights the importance of selecting appropriate menstrual suppression within this patient population. Not only are these patients at an increased risk of bleeding but they also experience an increased risk of venous thromboembolism (VTE) [6, 7]. Many variables can impact the risk of VTE including cancer type, presence of mediastinal mass, use of pro‐thrombotic chemotherapy agents, and the use of central venous catheters.

In addition to the clinical impact described, heavy menstrual bleeding has a significant psychological and emotional impact on patients. In a qualitative study, 20 AYA women with cancer undergoing chemotherapy and at risk for heavy menstrual bleeding were interviewed regarding their perceptions of menstrual suppression. Common themes that emerged included negative feelings and worry about menstrual bleeding, positive attitudes toward menstrual suppression, misconceptions about menstrual health, and a desire for tailored discussions about menstrual suppression [8]. Although menstrual suppression is a critical component of caring for patients with hematologic malignancies, there remain gaps in understanding and management. A single‐center retrospective cohort study characterizing menstrual suppression prescribing practices among pediatric and adult oncologists revealed that although the majority (77.8%) of patients received menstrual suppression, there was heterogeneity in the agents that were used and differing practices among pediatric and adult teams with regard to documentation of gynecologic course [9]. Moreover, in a survey of providers from 100 pediatric oncology centers, more than half agreed that patients with acute leukemias and lymphomas should receive menstrual suppression, and 95% felt it is important to consider menstrual suppression and that a formal guideline would be helpful. However, only 46% felt comfortable personally managing menstrual suppression [10].

Formalized guidelines often suggest risk‐versus‐benefit considerations, likely due to the interplay between bleeding and clotting risk factors leading to increased complexity [11]. The purpose of this review is to discuss pharmacologic menstrual suppression options in patients with hematologic malignancies and develop a systematic approach for agent selection based on patient‐specific risk factors.

## 2 Risk of Bleeding and Clotting With Hematologic Malignancies

Bleeding is a common complication experienced by patients with hematologic malignancies. A post hoc analysis of the A‐TREAT trial which included 330 patients with hematologic malignancies and hypoproliferative thrombocytopenia from therapy and/or underlying disease demonstrated that 91% of patients experienced bleeding of any World Health Organization (WHO) grade (Table 1), and 46% of patients experienced the WHO grade ≥ 2 bleeding [4]. When bleeding in the A‐TREAT trial was analyzed by body site, 37% of women of childbearing potential experienced vaginal bleeding of any WHO grade, and 28% experienced the WHO grade ≥ 2 vaginal bleeding despite widespread use of hormonal suppression [3].

**TABLE 1:** World Health Organization (WHO) bleeding scale [4, 12].

Grade 0	No bleeding
Grade 1	Petechiae
Grade 2	Mild blood loss
Grade 3	Gross blood loss (requiring transfusion)
Grade 4	Debilitating blood loss (potentially fatal)

The mechanisms of bleeding in patients with hematologic malignancies are multifactorial. Thrombocytopenia is a driver of bleeding and can occur as a result of the malignancy itself or secondary to the chemotherapy used to treat the malignancy [6]. Patients with relapsed or refractory hematologic malignancies may have a higher risk of bleeding compared to newly diagnosed patients, since they may be heavily pretreated. Additional risk factors for bleeding include fever or infection, indwelling catheters, procedures, and certain medications. As a result, menstruation and vaginal bleeding may be heavier or last longer in thrombocytopenic patients, leading to significant morbidity in patients who menstruate [13, 14].

Patients with hematologic malignancies not only have a higher risk of bleeding but they also have a higher risk of clotting compared to the general population. A large Danish population‐based cohort study found that, in the first 6 months following diagnosis, the incidence of VTE was 1.60% for acute myeloid leukemia (AML), 6.94% for acute lymphoblastic leukemia (ALL), 2.88% for Hodgkin lymphoma, and 2.66% for non‐Hodgkin lymphoma. In comparison, the incidence of VTE was 0.19% in the non‐cancer cohort [6, 7]. Variability in patient characteristics, chemotherapy regimens, and thromboprophylaxis may have impacted the incidences reported in this study.

The choice of chemotherapy regimen may also increase thrombosis risk. For example, asparaginase is a common component in treatment protocols for acute lymphoblastic leukemia that is associated with a higher incidence of VTE [15]. Additional risk factors that may increase thrombosis risk include prolonged immobilization, age, infections, presence of central venous devices, and cardiovascular disease [16].

As select menstrual suppression agents increase VTE risk, patient‐specific risk–benefit profiles must be considered prior to therapy selection.

## 3 Prophylactic Menstrual Suppression

### 3.1 Approach to Menstrual Suppression Agent Selection

Options for menstrual suppression include gonadotropin‐releasing hormone agonists (GnRH‐a), progestin‐only therapy, and combined hormonal contraception [11]. Each class of medications carries advantages and disadvantages, requiring clinicians to consider the following patient‐specific factors when selecting a menstrual suppression regimen:

- Presence of acute uterine bleeding (AUB)
- Desire for contraception
- Presence of VTE risk factors
- Degree of thrombocytopenia and neutropenia

Prophylactic menstrual suppression ideally starts prior to chemotherapy initiation in order to prevent heavy menstrual bleeding during periods of thrombocytopenia. Acute uterine bleeding, which is discussed later in this review, requires emergent treatment with multidose regimens of hormonal therapies. Given differences in the time required to achieve bleeding reduction or amenorrhea, some menstrual suppression agents cannot be used to treat emergent AUB. Additionally, some menstrual suppression agents are also contraceptives; however, this is not true for all agents. If a menstrual suppression agent that does not inherently provide contraception is chosen, an additional agent must be added if the patient desires contraception. Some menstrual suppression agents contain estrogen and may increase risk of VTE. As such, the decision to initiate an estrogen‐containing regimen warrants careful consideration of contraindications and risk factors and a risk–benefit discussion with the patient and care team. Finally, thrombocytopenia and neutropenia may increase bleeding and infection risk which impacts the timing of initiation of certain menstrual suppression agents such as insertion of an intrauterine device. A comparison of prophylactic menstrual suppression agents discussed in this section is found in Table 2.

**TABLE 2:** Comparison of prophylactic menstrual suppression agents.

Agent/Class	Dosing	Amenorrhea rate	Advantages	Disadvantages
Leuprolide [17]	3.75 mg monthly or 11.25 mg every 3 months IM	73%–96% [5, 18]	Most effective Long‐acting, improved adherence	No contraception Vasomotor symptoms Lower bone density Longer time to amenorrhea
CHC [19]	Ethinyl estradiol 20–35 mcg PO with second generation progestin daily	68%–88% [20]	Convenient, daily dosing	Increased VTE risk Adherence with daily dosing
DMPA [21]	150 mg IM or 104 mg SubQ every 3 months	50% at 1 year, 70% at 2 years [22, 23]	Long‐acting, improved adherence	Weight gain Lower bone density Longer time to amenorrhea Breakthrough bleeding, improves over time
LNG‐IUD [11]	Multiple available, replace every 3–8 years	20% at 1 year, 50% at 2 years [24, 25]	Long‐acting, improved adherence	Bleeding and infection risk on insertion Longer time to amenorrhea
POP [11]	MPA 10–20 mg/day PO NETA 5–15 mg/day PO	Up to 76% [26]	Convenient, daily dosing	Requires strict adherence Breakthrough bleeding, improves over time

Abbreviations: CHC, combined hormonal contraceptive; DMPA, depot medroxyprogesterone acetate; IM, intramuscular; LNG‐IUD, levonorgestrel intrauterine device; MPA, medroxyprogesterone acetate; NETA, norethindrone acetate; PO, by mouth; POP, progestin‐only pill; SubQ, subcutaneous; VTE, venous thromboembolism.

### 3.2 Gonadotropin‐Releasing Hormone Agonists

GnRH‐a have demonstrated amenorrhea rates of 73%–96% [5, 18]. In a study including 30 premenopausal women with hematologic malignancies eligible for stem cell transplant, all patients were treated with leuprorelin acetate 3.75 mg subcutaneously at least 30 days before their conditioning regimens and 28 days after the initial first dose. All but one patient (96.5%) were free of bleeding during the period of thrombocytopenia after transplant. Twenty‐eight patients were followed for a median of 17 months (range 3–51) and all developed amenorrhea [27].

Additionally, GnRH‐a have demonstrated superior efficacy in oncology patients compared to depot medroxyprogesterone acetate (DMPA) for menstrual suppression, which have demonstrated amenorrhea rates of 50%–70% at 12–24 months [22, 23]. In a retrospective review of 101 female oncology patients with regular menstrual cycles undergoing myelosuppressive treatments resulting in platelet counts below 25 k/μL, patients received either a GnRH‐a, DMPA, or no treatment for menstrual suppression. At the end of the study period, 76.9% of patients in the GnRH‐a group experienced amenorrhea compared to 45.2% of patients in the DMPA group. Moreover, no patients in the GnRH‐a group experienced moderate and severe vaginal bleeding compared to 21.4% who received DMPA and 40% of the untreated patients. None of the GnRH‐a‐treated patients required urgent treatment with conjugated estrogens to stop severe menorrhagia whereas it was needed in both the DMPA and untreated groups [28].

In addition, leuprolide administered with or without combined hormonal contraceptives (CHC) has shown reductions in RBC and platelet transfusion requirements in patients undergoing hematopoietic stem cell transplantation (HSCT). A retrospective cohort study including 214 patients with AML, ALL, or other hematologic malignancies studied whether leuprolide, leuprolide with CHC, or CHC alone administered before HSCT minimized transfusion requirements within 90 days post‐HSCT compared to no treatment. Leuprolide with or without CHC demonstrated statistically significant reductions in RBC and platelet transfusions compared to no treatment, and numerical reductions in transfusions compared to CHC alone. The incidence of VTE did not differ between groups. Notably, the leuprolide groups and CHC alone group had similar rates of abnormal uterine bleeding in the post‐HSCT period, ranging from 32% to 52%; however, the definition of abnormal uterine bleeding was not defined, preventing conclusions regarding severity of bleeding [29].

GnRH‐a may be considered first line for menstrual suppression in AYAs with hematologic malignancies due to their high rates of efficacy; however, they must ideally be initiated several weeks prior to the start of chemotherapy due to the initial flare response, characterized by an increase in release of follicle‐stimulating hormone (FSH) and luteinizing hormone (LH) and downstream increase in estrogens and other hormones [11]. This can cause a temporary worsening of symptoms, including heavier bleeding. The initial flare response typically lasts 2–3 weeks after the first dose. Over time, the hypothalamic–pituitary–ovarian (HPO) axis becomes desensitized to GnRH, resulting in a low‐estrogen state. Given the time needed to achieve treatment effect, GnRH‐a should not be used to treat AUB. Adverse effects of GnRH‐a include vasomotor symptoms (VMS) and bone density loss [17]. Additionally, GnRH‐a are not a form of contraception. If a patient desires contraception, an additional agent such as a CHC or progestin must be added.

Although the mechanism of action of GNRH‐a involves changes in circulating FSH and LH levels, routine monitoring of FSH and LH levels is not recommended due to limited utility in clinical decision‐making [30]. In patients in which the efficacy of the chosen menstrual suppression regimen is in question, collaboration with obstetrics and gynecology colleagues may be considered to guide monitoring.

There is limited evidence to suggest an increased likelihood of ovarian preservation and pregnancy in patients with cancer treated with gonadotoxic chemotherapies who receive concomitant GnRH‐a compared to those who do not receive GnRH‐a. Most trials suggesting benefit were conducted in patients with breast cancer [31]. Studies in patients with hematologic malignancies are small and have not demonstrated benefit [32]. Current guidelines recommend that GnRH‐a may be offered as an adjunct fertility preservation method for females with breast cancer but should not be used in place of established fertility preservation methods such as oocyte, embryo, or ovarian tissue cryopreservation. A full review of fertility preservation is outside the scope of this review. For current recommendations, refer to the American Society of Clinical Oncology guidelines [31].

### 3.3 Combined Hormonal Contraceptives

CHCs have demonstrated amenorrhea rates of 68%–88% when dosed continuously and skipping placebo pills [20]. Despite the high rates of efficacy, CHCs must be used with caution in patients with hematologic malignancies given the increased risk of VTE.

In the general population, use of CHC increases the risk of VTE approximately threefold to sixfold compared to non‐users. Higher doses of ethinyl estradiol in CHC are associated with higher VTE risk. Additionally, third‐ and fourth‐generation progestins, such as desogestrel and drospirenone, respectively, carry a higher risk of VTE compared to second‐generation progestins, such as levonorgestrel [33]. A meta‐analysis of VTE risk with CHCs assessed the risks with different progestins and estrogen doses. When compared to the reference standard of levonorgestrel and similar doses of ethinyl estradiol, the relative risk of VTE was 1.46 (95% confidence interval (CI): 1.33–1.59) with desogestrel/ethinyl estradiol and 1.4 (95% CI: 1.26–1.56) with drospirenone/ethinyl estradiol [19]. The incidence of VTE in patients with hematologic malignancies using CHCs has not been quantified. As such, clinicians must consider general and oncology‐related risk factors and contraindications prior to the initiation of CHC in this patient population. CHCs should be avoided in patients with contraindications to estrogen use, including oncology‐specific contraindications (Table 3) [34]. CHCs should be used with caution in patients with VTE risk factors following a risk–benefit discussion with the patient and care team. Patients with no additional risk factors can be considered for use of CHCs for menstrual suppression. Additionally, CHCs provide contraception for patients who desire it. CHCs also have a place in therapy for AUB given their quick onset of action.

**TABLE 3:** Contraindications to estrogen use [34].

General contraindications	Oncology‐related contraindications
Migraine with aura Age ≥ 35 y and smoking ≥ 15 cigarettes/day History of VTE with high risk of recurrence Hepatic dysfunction and/or liver cancer	Receiving concomitant therapy or supportive agents that increase thromboembolic risk (e.g., asparaginase, erythropoiesis‐stimulating agents) Presence of mediastinal mass

Abbreviation: VTE, venous thromboembolism.

### 3.4 Depot Medroxyprogesterone Acetate

Depot medroxyprogesterone acetate is a long‐acting progestin that provides amenorrhea rates of 50%–70% at 12–24 months in the general population [22, 23]. As discussed previously, DMPA is a less effective option compared to GnRH‐a to reduce vaginal bleeding in women undergoing myelosuppressive chemotherapy [28]. DMPA is provided as either an intramuscular or subcutaneous injection every 3 months. In periods of severe thrombocytopenia, intramuscular administration of DMPA may increase the risk of intramuscular hematoma, and subcutaneous dosing is preferred. Breakthrough bleeding may occur earlier in therapy but appears to improve over time. One common side effect of DMPA is weight gain. Additional risks include reduction in bone mineral density (BMD) due to a low estrogen state [21]. The loss of BMD associated with DMPA use may be irreversible, and it currently carries a black boxed warning to avoid long‐term use for more than 2 years. This is an especially important consideration for patients undergoing HSCT and in AYAs who are receiving corticosteroids as part of treatment for ALL. HSCT‐associated bone loss is experienced by approximately 24%–60% of patients between 2 and 12 months after transplant, and steroid‐induced osteonecrosis has a reported prevalence of up to 39% with female sex, which is a notable risk factor [35, 36].

DMPA is a form of contraception and is a favorable option in patients seeking contraception and desiring to avoid daily dosing. For menstrual suppression, DMPA is not as effective as other agents but is an option in patients who cannot use leuprolide. However, given that DMPA provides contraception, it represents an option that can be added to leuprolide and can provide additional reduction in menstruation.

### 3.5 Levonorgestrel Intrauterine Device

Rates of amenorrhea approach 20% at 1 year and up to 50% at 2 years after insertion of the levonorgestrel intrauterine device (LNG‐IUD) in the general population [24, 25]. The LNG‐IUD is also approved by the United States Food and Drug Administration (FDA) for nonacute abnormal uterine bleeding and demonstrates efficacy for the treatment of heavy menstrual bleeding (HMB). A meta‐analysis of 21 randomized controlled trials compared the LNG‐IUD versus no treatment, placebo, or medical or surgical therapy for HMB in women of reproductive age. Compared to placebo, HMB was 99.5% lower in the LNG‐IUD group at 6 months. Compared to oral therapies, including CHCs and progestin‐only pills (POPs), the reduction in blood loss was 55%–67% improved in patients treated with the LNG‐IUD [37]. Of note, there is significant heterogeneity in the dosing of the CHCs and POPs used in the studies evaluating HMB, which differ from the dosing recommended for menstrual suppression.

LNG‐IUDs are often not able to be inserted immediately following the diagnosis of a hematologic malignancy. Patients frequently present with thrombocytopenia, which increases the risk of bleeding upon insertion, and neutropenia, which increases infection risk. LNG‐IUDs may be continued if placed prior to diagnosis or placed between chemotherapy cycles when blood counts recover. LNG‐IUDs provide contraception for patients as well as convenience since they can remain in place for years. Although amenorrhea has been shown to occur in the general population, rates are lower than other menstrual suppression agents and there are no direct studies in patients with hematologic malignancies. They can also be used in combination with a GnRH‐a to provide contraception.

### 3.6 Progestin‐Only Pills

Oral POPs may be used for menstrual suppression; however, the rates of amenorrhea may be lower compared to the other options mentioned. A small study of 44 women with intellectual and developmental disabilities who were treated with POPs reported amenorrhea rates of up to 76% at 2 years [26]. Commonly used regimens for menstrual suppression include medroxyprogesterone acetate (MPA) 10–20 mg/day and norethindrone acetate (NETA) 5–15 mg/day [11]. Of note, oral MPA and NETA are not FDA‐approved for contraception. However, MPA and NETA are theorized to provide contraception due to their mechanisms of action. Pharmacokinetic data also demonstrate higher norethindrone levels in patients taking NETA 5 mg/day compared to the standard norethindrone 0.35 mg/day used for contraception [38]. Other POPs, including drospirenone 4 mg/day and norethindrone (NET) 0.35 mg/day, provide contraception but do not provide the same degree of amenorrhea and are not used for menstrual suppression [11]. Moreover, drospirenone is a fourth‐generation progestin and is associated with a higher VTE incidence compared to earlier generation progestins. POPs may be considered in patients who are ineligible for CHC due to thromboembolic risk and prefer an oral menstrual suppression regimen. Limitations include the requirement for strict adherence in order to be effective as contraception. Additionally, POPs may be used for AUB given their quick onset of action.

## 4 Management of Acute Uterine Bleeding

Patients may initially present or develop AUB requiring aggressive treatment. Multi‐dose regimens of CHCs or POPs (MPA or NETA) are considered first line for treatment of AUB [11]. Both regimens are considered equally effective; however, POPs are preferred in patients with thromboembolic risk factors. A prospective, open‐label trial randomized 40 hemodynamically stable patients with AUB sufficient to justify immediate medical or surgical intervention to either MPA 20 mg three times daily for 1 week followed by 20 mg daily for 3 weeks or ethinyl estradiol 35 mcg‐norethindrone 1 mg three times daily for 1 week followed by once daily for 3 weeks. The results showed no difference between groups in cessation of bleeding or avoidance of emergent procedures. Days to bleeding cessation were approximately 3 days with both regimens [39]. It is important to note that dosing of CHCs and POPs for AUB is more frequent compared to dosing used for prophylactic menstrual suppression. For example, MPA 10–20 mg/day is used for menstrual suppression, whereas doses up to 60–80 mg twice daily may be used for AUB. Similarly, NETA 5–15 mg/day is used for menstrual suppression, whereas doses up to 10–20 mg every 8 h may be used for AUB. For CHCs, a monophasic pill with 30–50 mcg ethinyl estradiol with a progestin is given every 6–8 h for AUB, whereas it is taken once daily for menstrual suppression [11]. Once bleeding attenuates or resolves, multidose CHCs and POPs must be tapered over several days as tolerated to maintain control of bleeding, and a maintenance menstrual suppression regimen should be initiated. CHCs or POPs may be continued for menstrual suppression if there are no contraindications to treatment. Patients may also be transitioned to other menstrual suppression agents discussed above.

If bleeding does not resolve with CHCs or POPs alone, antifibrinolytic agents such as tranexamic acid (TXA) 1.3 g three times daily may be added as an adjunctive agent for up to 5 days [11, 40]. TXA is FDA approved for the treatment of chronic abnormal uterine bleeding and has demonstrated a mean reduction in menstrual blood loss of approximately 70 mL or 40% [41]. TXA has not been studied for emergent uterine bleeding but is presumed effective for this indication as well. TXA is associated with an increased risk of thromboembolism in the general population; however, there does not appear to be an increased risk of thrombosis in patients with hematologic malignancies treated with TXA. The A‐TREAT trial randomized patients with hematologic malignancies to prophylactic TXA or placebo to determine whether there was a difference in incidence of the WHO grade ≥ 2 bleeding. Reported safety outcomes showed no significant difference in thrombotic events between study groups (6 events [3.7%] in the TXA group and 9 events [5.5%] in the placebo group) [4].

There is insufficient evidence to support the use of megestrol for the management of AUB in patients with hematologic malignancies. A small case series including six adolescent patients at a single institution who received megestrol acetate for abnormal uterine bleeding demonstrated that bleeding stopped in five of six patients after receiving megestrol; however, four of six patients also received concomitant estrogen [42]. In addition to a paucity of efficacy data, there remains a question of safety in patients with hematologic malignancies. Megestrol is associated with an increased risk of VTE, but it is unclear whether the same degree of risk is present in patients with hematologic malignancies. In a trial of patients with cancer‐related anorexia/cachexia randomized to megestrol, dexamethasone, or fluoxymesterone, megestrol trended toward a higher rate of deep vein thrombosis (DVT) compared to dexamethasone (5% vs. 1%; *p* = 0.06) [43]. A retrospective review of 97 oncology patients who were prescribed megestrol for cachexia detected 11 VTEs after megestrol use, with four of these occurring in pancreatic cancer cases. The authors concluded that pancreatic cancer, chemotherapy regimens, and megestrol may have contributed to thrombotic risk. Given the unclear thrombotic risk of megestrol and the presence of other options to treat AUB, megestrol should be avoided in this patient population for treatment of AUB [44]. A summary of options for management of prevention and management of vaginal bleeding is found in Table 4.

**TABLE 4:** Summary of agents for prevention and treatment of vaginal bleeding.

	Acute bleeding	Contraception	Amenorrhea	VTE risk
GnRH‐a			+++	
CHC	✔	✔	+++	+++
DMPA		✔	++	
LNG‐IUD		✔	++	
MPA/NETA	✔	✔^a^	+	+
TXA	✔			

Abbreviations: CHC, combined hormonal contraceptive; DMPA, depot medroxyprogesterone acetate; GnRH‐a, gonadotropin‐releasing hormone agonist; LNG‐IUD, levonorgestrel intrauterine device; MPA, medroxyprogesterone acetate; NETA, norethindrone acetate; TXA, tranexamic acid; VTE, venous thromboembolism.

^a^ MPA/NETA are theorized to provide contraception due to their mechanisms of action. Pharmacokinetic data demonstrate higher norethindrone levels in patients taking NETA 5 mg/day compared to the standard norethindrone 0.35 mg/day used for contraception [38].

## 5 Patient Case #1

A 23‐year‐old female presents to an academic medical center with a new diagnosis of acute lymphoblastic leukemia (ALL). She will begin induction chemotherapy, which includes cyclophosphamide, daunorubicin, vincristine, peg‐asparaginase, and steroids. She has no other significant past medical history, and her only prior‐to‐admission medication was norgestimate 0.25 mg‐ethinyl estradiol 35 mcg daily for contraception. The team is interested in initiating a menstrual suppression regimen concurrently with her induction, and the patient expresses she is interested in a menstrual suppression regimen that also provides contraception. Which menstrual suppression regimen do you recommend?

It would be unsafe for the patient to continue her CHC given the increased VTE risk with peg‐asparaginase. Leuprolide is a safe and effective option for this patient, with high rates of amenorrhea in this patient population. Onset of menstrual suppression with leuprolide will take several weeks. Use of POPs can be considered for any AUB that occurs. Leuprolide does not provide contraception on its own, so the addition of LNG‐IUD, DMPA, or chronic POP would need to be considered. LNG‐IUD placement may not be available while admitted but represents an option upon discharge and once blood counts have recovered from chemotherapy.

## 6 Patient Case #2

A 36‐year‐old female with a past medical history of lower extremity DVT (not on anticoagulation), transient ischemic attack, and newly diagnosed AML presents for induction chemotherapy with daunorubicin and cytarabine. On Day 10 of induction, she begins to experience AUB, requiring one pad per hour. What pharmacologic recommendations would you make to treat this patient?

This patient is not a candidate for estrogen‐based therapy to attenuate bleeding given her history of DVT, transient ischemic attack, and risk of VTE recurrence in the setting of malignancy. As such, appropriate initial therapy should consist of multidose progestin‐based regimens, such as NETA 10–20 mg every 8 h or MPA 60–80 mg twice daily until bleeding stops, then tapering every few days. Once the acute phase has resolved, it is imperative to initiate a maintenance menstrual suppression regimen to prevent future episodes.

## 7 Conclusion

Caring for menstruating patients with hematologic malignancies requires an understanding of the prevention and treatment of vaginal bleeding. Despite the recognition of the need for menstrual suppression in this patient population, gaps remain in the literature and in patient care. Challenges to conducting robust, prospective, large‐scale, randomized controlled trials include heterogeneity in malignancies, variable thrombotic risk factors, multiple changes in suppression regimens, and privacy concerns with pediatric populations. To address current gaps, future studies could randomize patients across a spectrum of thrombosis risk to receive either CHCs or POPs for menstrual suppression to determine the risk of thrombosis in this patient population and quantify whether the presence of hematologic malignancy and certain risk factor(s) increases thrombosis risk.

Based on the available body of literature, the presence of AUB, presence of VTE risk factors, desire for contraception, and degree of thrombocytopenia and neutropenia are all factors that impact agent selection for menstrual suppression in patients with hematologic malignancies. If considering the use of CHC for menstrual suppression, it is important to consider absolute contraindications and thromboembolic risk‐enhancing factors to inform risk–benefit discussions with the patient and care team. Providers must recognize the nuances of each menstrual suppression agent and consider patient‐specific characteristics to select the optimal regimen and continually adjust based on patient response, side effects, and preferences.

## Author Contributions

**Sahaana Veeravalli:** investigation, writing – original draft, writing – review and editing. **Allison Schepers:** conceptualization, writing – review and editing, investigation, writing – original draft. **Lydia L. Benitez:** conceptualization, writing – review and editing, investigation, writing – original draft. **Martina S. Fraga:** conceptualization, writing – review and editing, investigation, writing – original draft.

## Funding

The authors have nothing to report.

## Conflicts of Interest

The authors declare no conflicts of interest.

## Data Availability

Data sharing is not applicable to this article as no datasets were generated or analyzed during the current study. All information supporting this review is available within the article and the cited references.
